# Clinical and Electromyographic Assessment of Swallowing in Individuals with Functional Dysphonia Associated with Dysphagia Due to Muscle Tension or Atypical Swallowing

**DOI:** 10.3390/audiolres11020015

**Published:** 2021-04-13

**Authors:** Paulina Krasnodębska, Agnieszka Jarzyńska-Bućko, Agata Szkiełkowska, Jędrzej Bartosik

**Affiliations:** 1 Audiology and Phoniatrics Clinic, Institute of Physiology and Pathology of Hearing, 02-042 Warsaw, Poland; a.jarzynska@ifps.org.pl (A.J.-B.); a.szkielkowska@ifps.org.pl (A.S.); 2 Audiology and Phoniatrics Faculty, Fryderyk Chopin University of Music, 00-368 Warsaw, Poland; 3 Otorhinolaryngology Clinic, Institute of Physiology and Pathology of Hearing, 02-042 Warsaw, Poland; bartosikjedrzej@gmail.com

**Keywords:** dysphagia, dysphonia, muscle tension dysphagia, swallowing, muscle tension dysphonia, electromyography

## Abstract

Introduction: Over the past few years, attention has been paid to the coexistence of dysphonia with dysphagia, in the context of functional disorders. The aim of this work was to objectify logopaedic examination of dysphonic patients with coexisting swallowing difficulties by surface electromyography. Methods: The material of the work included 58 patients with muscle tension dysphonia (MTD). Each patient underwent otolaryngologic, phoniatric and logopaedic examination. We collected information about medical history and asked patients to fill out Reflux Symptom Index (RSI), Eating Assessment Tool (EAT-10), Dysphagia Handicap Index (DHI) and Swallowing Disorder Scale (SDS). The algorithm of dysphagia diagnostics in our clinic assumes parallel surface electromyography (SEMG) during Functional Endoscopic Evaluation of Swallowing. Results: In comparison to patients suffering from atypical swallowing, patients with muscle tension dysphagia (MTDg) obtained higher values from almost all questionnaires. Logopaedic evaluation revealed abnormalities in the structure and efficiency of the articulatory organs and in the assessment of primary functions. Patients with more abnormalities in logopaedic examination had significantly higher infrahyoid muscle activity during swallowing observed in EMG. Patients with non-normative swallowing pattern had significantly greater asymmetry of the average and maximum amplitude of masseters, as well as submental muscles. Patients with higher percent of muscles asymmetry gained higher scores in questionnaires. Conclusions: Surface electromyography objectifies logopaedic examination of patients with swallowing difficulties. The results of this work showed that, apart from longer swallows, patients with MTDg differ from patients with non-normative swallowing patterns in the muscle activity measured by SEMG, abnormalities in logopaedic evaluation and the severity of complaints reported by patients.

## 1. Introduction

Over the past few years, attention has been paid to the coexistence of dysphonia with dysphagia, especially in the context of functional disorders. In 2016, Kang added the term Muscle Tension Dysphagia (MTDg) to dysphagia nomenclature that completed the spectrum of the disease [1]. This dysphagia subtype is defined as a type of laryngeal muscle tension disorder manifesting primarily as swallowing difficulty with or without any accompanying organic cause, laryngeal hyperresponsiveness and/or nonspecific laryngeal inflammation. Kang distinguished two types of MTDg. Primary-without any contributing causes and secondary-with accompanying organic cause, laryngeal hyperresponsiveness and/or nonspecific laryngeal inflammation. Epidemiology of the disorder reported by researchers shows that MTDg is diagnosed in about 5% of patients hospitalised in otorhinolaryngology (ENT) departments [2]. Another research reports the occurrence of MTDg in 4% of patients hospitalised in the phoniatric department due to voice dysfunction [3]. The differential diagnosis of MTDg should include, first and foremost, non-normative swallowing pattern (atypical swallowing). This type of swallowing dysfunction is not considered as a dysphagia subtype. The term is found in speech therapy nomenclature rather than otolaryngological nomenclature. Patients with atypical swallowing may report a sensation of delay, without having an objective symptomatology regarding the duration of swallowing phases, while in patients with MTDg, one can observe objective delay in transit of a liquid or solid bolus.

The concept of the above-mentioned swallowing difficulties can be further defined according to Jalil’s definition of subjective and objective dysphagia [4]. In 2015, the author defined objective dysphagia as an abnormal delay in transit of a liquid or solid bolus during the oropharyngeal or esophageal stages of swallowing. MTDg meets the criteria of objective dysphagia. Jalil defined subjective dysphagia as a sensation of a delay in transit of a liquid or solid bolus during the oropharyngeal and/or esophageal stages of swallowing. Non-normative swallowing pattern meets the criteria of subjective dysphagia. Atypical swallowing is diagnosed mainly by speech therapists. Non-normative swallowing pattern results from abnormal motility of the tongue, lips, malocclusion, nonphysiological breathing pattern or other parafunctions within the masticatory apparatus. The type of dysfunction carries no greater risk of aspiration of the digestive content than in the healthy population [5].

Normative values of the duration of a swallow were, in detail, developed by Vaiman and co-authors in a series of studies based on electromyographic examinations [6,7,8]. Before those publications, normative values of the oropharyngeal phase duration were based on videofluoroscopy. Vaiman et al. observed that the duration of one swallow of water lasted 1–5.74 s, and the duration of 20 cc swallowed lasted 1.8–6.2 s for patients aged 18–70 years. Patients aged more than 70 years had a longer duration of the swallow, 2.3–6.7 and 1.8–8.13 s, respectively.

It should be pointed out that, apart from dysphagia diagnostic procedure based on endoscopic examinations, extended logopaedic evaluation plays an extremely important role in case of dysphagia symptoms reported by a patient. The literature study shows that nearly 80% of patients with voice disorders who reported difficulties during swallowing were diagnosed with dysphagia (including MTDg) or non-normative swallowing pattern [3].

### Aim

The aim of this work was to objectify logopaedic examination of patients with swallowing difficulties by surface electromyography (SEMG). Specific objectives concerned the correlation of logopaedic evaluation of muscle function with SEMG parameters and the severity of complaints reported by patients. The second aim applied the analysis of the results in the context of muscle tension dysphagia and atypical swallowing differentiation.

## 2. Material and Method

The material of the work included 58 patients with muscle tension dysphonia (MTD) hospitalized in the Audiology and Phoniatric Clinic in 2018 due to reported swallowing dysfunction and who were diagnosed during the stay with muscle tension dysphagia (32 patients) or non-normative swallowing pattern (26 patients). The study design was approved by the local Bioethics Committee KB.IFPS.24/2017.

The group included 34 women and 24 men not older than 70, with the mean age of 62 years (standard deviation SD-11).

### 2.1. Assessment Methods

In the study, each patient underwent otolaryngological and phoniatric examination. We collected information about medical history and asked patients to fill out Reflux Symptom Index (RSI), Eating Assessment Tool (EAT-10), Dysphagia Handicap Index (DHI), Swallowing Disorder Scale (SDS) and Voice Handicap Index (VHI) [3]. The algorithm of dysphagia diagnostics in our clinic assumes parallel surface electromyography (SEMG) during Functional Endoscopic Evaluation of Swallowing (FEES). FEES was carried out using fibreoptic endoscope Olympus Evis Exera III CV 190. Nine attempts of food intake were evaluated: three with water (10, 15 and 20 mL), three with yogurt (10, 15 and 20 mL) and three with the bread roll (3, 6 and 9 g). The time necessary for effective swallowing and chewing and the number of swallows were measured. Moreover, patients were evaluated with the Reflux Finding Score (RFS) [9].

Patients were diagnosed with MTDg, based on Kang’s and Jalil’s definitions of dysphagia [1,4]. We decided to diagnose MTDg in patients with excessive throat and larynx muscle tension observed during swallowing, who reported swallowing complaints and had objective delay in the transport of liquid or solid substances during the oropharyngeal phase of swallowing (as the sole abnormality of FEES). Non-normative swallowing pattern was diagnosed based on speech therapist examination in patients with normal results of FEES. Those patients did not present objective delay in the transport of liquid or solid substances, as stated in the definition of objective dysphagia by Jalil [4].

#### 2.1.1. Surface Electromyography (SEMG)

We used Neurosoft 4 channel EMG device to examine simultaneously symmetrical muscles (masseter, trapezius, submental and infrahyoid). The selection of muscles for the analysis was based on the work of Vaiman et al. [6,7,8]. The decision of trapezius inclusion was inspired by observations of significantly increased trapezius muscle tension in patients with muscle tension dysphonia [10]. The examination was performed by a physician, a phoniatrist certified in electrodiagnostic medicine, and analysed together with a clinical neurophysiologist. Surface electromyography was made using standard surface sensors. Skin under the electrodes was lightly scrubbed with alcohol gauze pads (if necessary, peeling was used). Electric impedance was measured before the examination and did not exceed 4µV. Concentric surface disc electrodes with applicated electrode gel were attached symmetrically to patients’ skin above the masseters. Cup electrodes with electrolytic gel were adhered above the remaining examined muscles: submental, beneath the chin about 1.5 cm from the midline; infrahyoid, on both sides of the thyroid cartilage; and trapezius muscles, 7 cm from the midline. Surface disk ground electrode was adhered in the middle of the forehead and the reference electrode on the nasal bridge.

As stated by Vaiman, SEMG evaluation is limited to timing and amplitude measurements [8]. Based on the Neurosoft software, we analysed the subsequent parameters of the SEMG recording: mean and maximum amplitude, symmetry of amplitude of the examined pairs of muscles (expressed as a percentage of the ratio of the mean amplitude of one muscle to the sum of the mean amplitudes of both muscles). Muscle activity was quantified in microvolts. Drawing upon the research conducted by Wang, we defined the percentage of amplitude reduction <20% as normal [11].

#### 2.1.2. Logopaedic Evaluation

A speech therapist assessed the anatomical structure and efficiency of the oral organs: tongue (including palpation of sublingual frenulum; coordination of tongue movements, retraction and lateral movements necessary for oral processing and transport of bite and possible retention in the oral cavity or vestibule of the mouth); hard and soft palate; masseter, cheeks, and submental muscle activity; lips and possible abnormalities in their closing; jaw mobility; dentition and temporo–mandibular joints [5]. If a masseter had >20% amplitude difference measured by SEMG, there was obvious visible asymmetry reported by the speech therapist. The therapist evaluated the oral phase of swallowing and the coordination of the oral and pharyngeal phases. In selected cases, a retrospective analysis of various primary activities was carried out with emphasis on the development of these skills.

### 2.2. Statistical Analysis

For statistical analysis of parameters obtained in the work, the following tests were used: Pearson and Spearman correlation and Mann–Whitney test. The level of statistical significance was set at *p* < 0.05. The degree of measured correlation was classified according to the division: 0.0 ≤ |r| ≤ 0.2—no correlation; 0.2 <|r| ≤ 0.4—weak correlation; 0.4 < |r| ≤ 0.7—average correlation; 0.7 < |r| ≤ 0.9—strong correlation; 0.9 < |r| ≤ 1.0—very strong correlation [3].

## 3. Results

Patients reported subsequent factors of medical history: 16 patients had laryngopharyngeal reflux (LPR), 6 patients suffered from gastroesophageal reflux disease (GERD), 8 patients reported obstructive sleep apnoea syndrome (OSAS) and 2 patients reported eustachian tubes dysfunction. Table 1 shows the results of questionnaires in the two groups of patients (MTDg and atypical swallowing). The highest values obtained from almost all questionnaires were reported by patients with MTDg. Only VHI results were smaller in the group of patients with MTDg, in comparison with patients suffering from atypical swallowing. Statistically significant differences between the groups of patients were found for EAT-10, DHI and RSI results.

### 3.1. Results of Logopaedic Evaluation

Logopaedic evaluation revealed abnormalities in the structure and efficiency of the articulatory organs and in the assessment of primary functions in all examined patients. Table 2 shows the percentage of patients divided according to swallowing dysfunction cause, MTDg or non-normative swallowing pattern, presenting dysfunctions within the evaluated structures. The most commonly observed problems concerned tongue and dental defects. Limited mobility of the tongue was frequently observed as a result of incorrect structure of the sublingual frenulum. Dental defects included malocclusion, missing teeth (premolars and molars) and incorrectly fixed lower dentures. In nearly half of the patients, dysfunctions of the temporo–mandibular joint were reported, while mandible movement (dislocations of the joint discs without blockage, slipping and clicking or popping sound). According to the logopaedic examination all patients in both groups manifested dysfunction of the oropharyngeal stage of swallowing. Abnormal oral primary functions, such as biting, chewing and nonphysiological breathing, were observed in patients with MTDg (respectively, in 100%; 100%; 94%) and patients with atypical swallowing (respectively, in 69%; 85%; 69%).

Statistical analysis revealed a weak correlation of the sum of abnormalities in anatomical and functional logopaedic evaluation with the group type (0.36). The abnormalities were more often observed in patients with non-normative swallowing patterns. No correlation was found between the logopaedic evaluation (anatomical and functional or solely functional) and the results of questionnaires. The analysis of oral primary functions evaluation showed a correlation of average strength (0.63) with the group subtype. MTDg patients had significantly more parafunctions. We found no correlation of primary function occurrence and severity with the results of the questionnaires.

### 3.2. Observation of Swallowing during FEES

**Figure 1:**
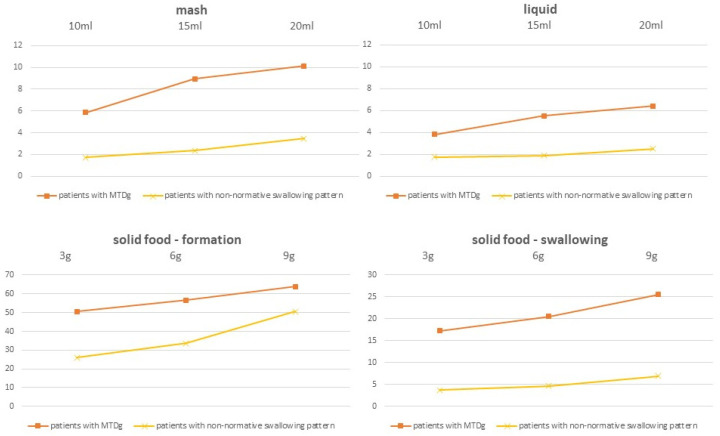
Duration of food swallowing and chewing in the subgroups of patients.

As mentioned previously by the authors of this article, one of the group’s differentiating criterion was the duration of swallow. We have adopted the results developed by Vaiman et al. as normative values used in Functional Endoscopic Evaluation of Swallowing (FEES) [6]. Figure 1 shows the duration of food swallowing and chewing in the subgroups of patients. The biggest differences between patients with MTDg and a non-normative swallowing pattern were visible during solid food, then mash, swallow. Moreover, the figure demonstrates a mild increase of duration of the activities in patients with the increase of food volume. Statistical analysis showed no relationship between the length of food swallowing and formation and the number of abnormalities (anatomical and functional or solely functional) assessed by the speech therapist. The analysis of oral primary functions evaluation showed a correlation of weak strength (0.49) with the duration of food swallowing and formation. Patients who swallowed and chewed longer (the strongest correlation with number of swallows of solid food) had significantly more parafunctions. We did not notice the increase of correlation strength with the increase of food volume. Statistically significant correlations were also found between the duration of food swallowing and formation of all kinds of food and the RSI score.

### 3.3. Results of SEMG

Surface electromyography allowed us to observe the examined muscles in real time in order to determine the strength, as well as the symmetry, of the recorded muscle activity. Table 3 shows the values of the mean and maximum amplitude and the differences between the same muscle pairs. Statistical analysis of the average and maximum amplitude values showed no significant relation with the group type. We found significant relation of logopaedic evaluation and amplitude values (average, as well as maximum) of the infrahyoid muscles measured during swallowing of all kinds of food. Patients with more abnormalities in logopaedic examination had significantly higher infrahyoid muscle activity during swallowing.

The average and maximum differences in amplitude between the examined submental, infrahyoid and trapezius muscles, on average, mostly did not exceed a dozen or so percent. The largest average differences between sides were observed in the recordings of the masseters. Statistical analysis of the value of amplitude asymmetry showed significant relation with the group type. Patients with non-normative swallowing patterns had significantly greater asymmetry of the average and maximum amplitude of masseters during mash and solid food swallowing and formation, as well as submental muscles during liquid swallowing (Table 4). As shown in Table 3, more than 50% of patients with MTDg and around 20% of patients with a non-normative swallowing pattern exceeded the value of 20% of masseter maximal amplitude asymmetry during swallowing and formation of all types of food. We observed an increased percentage of patients in the MTDg group who exceeded the value of 20% of submental maximal amplitude asymmetry during swallowing and formation of all types of food.

Statistical analysis of the value of amplitude asymmetry showed significant relation with logopaedic evaluation. Patients with higher asymmetry of average and maximum amplitude measured above the infrahyoid muscles during liquid and mash swallowing, as well as patients with higher asymmetry of average amplitude measured above the trapezius muscles during solid food formation and liquid swallowing, presented more abnormalities in logopaedic examination.

We observed longer durations of solid food formation and swallowing in patients with smaller average and maximum amplitude of the submental and infrahyoid muscles. Longer duration of swallowing of mash and solid food was also observed in patients with greater asymmetries of the average and maximum amplitudes of the submental and infrahyoid muscles (weak correlation), as well as the trapezius muscles (strong correlation).

#### Relation of SEMG and the Results of Questionnaires

Patients with higher percent of muscles asymmetry gained higher scores in questionnaires. Significant correlations were found between the submental muscles asymmetry during mash swallowing and the results of SDS, DHI and VHI; the infrahyoid muscle asymmetry during liquid swallowing and the results of EAT-10 and VHI; and the trapezius muscle asymmetry during solid food swallowing and the SDS, DHI, EAT-10 and VHI results.

The analysis of muscle amplitude with the severity of reflux symptoms expressed through the RSI score revealed a significant relation with the infrahyoid and submental muscles. Higher RSI results correlated with higher mean and maximum amplitudes of the infrahyoid muscles during mash and solid food swallowing and of the submental muscles during liquid swallowing. Moreover, we found significant relations of the infrahyoid muscles asymmetry during liquid and mash swallowing with the RFS score.

## 4. Discussion

Dysphagia mainly affects patients after stroke and is the domain of neurological departments, although it is increasingly of interest to otolaryngologists and phoniatricians. The problem of functional swallowing disorders associated with muscle dysfunction seems to be underestimated. In logopaedic nomenclature, it is defined as non-normative swallowing pattern. The term is not considered as a dysphagia subtype. It means a disfunction of swallowing, rather than disorder. Kang noticed the importance of the above-described problem and introduced a new term: Muscle Tension Dysphagia [1]. Moreover, she started a discussion about the need for new diagnostic and therapeutic perspectives in this group of patients. The results of further works on MTDg allowed for a more detailed definition. The results of our research show that patients with MTDg not only differ in swallowing duration (which should be included in the definition) but also in SEMG characteristics.

Literature data shows coexistence of dysphagia in about 5–10% of patients with voice disorders [2,3,12]. As mentioned above, the problem seems to be underestimated. Screening of dysphagia in patients with voice disorders should include simple tasks. Logopaedic procedure ought to be a key element not only in the treatment but also primary diagnosis. The next screening tools should be questionnaires. Moreover, the results of this work confirm that SEMG is a reliable, noninvasive, time-saving and inexpensive procedure providing information on the timing of selected muscle contraction patterns during swallowing [7].

Review of the laryngeal electromyography (LEMG) literature showed that, initially, SEMG was not considered as an appropriate examination for laryngeal muscles [13]. Surface electrodes are noninvasive but the least selective electrode type, not suitable for recording details or electrical events associated with individual motor units. The recorded potential represents the weighted sum of all the individual potentials produced by the activated muscle, each divided by its respective distance from the electrode [14]. Despite its limitations, authors predicted that surface LEMG would play a larger role in the evaluation of speech and swallowing disorders as the technique improves [15]. Numerous researchers used SEMG in phonation studies to assess patients with MTDg [16]. A number of past studies have used surface electromyography of the suprahyoid muscles to assess oropharyngeal dysfunction in dysphagia [17,18]. Application of the method provided an alternative for videofluoroscopy, so far the only tool allowing the evaluation of the oropharyngeal phase of swallowing. Studies of Vaiman changed the unfavourable attitude of otolaryngologists towards LEMG and supplied the clinicians with a relatively simple tool and normative database for the evaluation of swallowing [8]. His surface EMG studies were performed on 440 normal adults. The researchers established normative data for the duration of muscle activity during swallowing and drinking for four muscle groups (orbicularis oris, masseter, submental, infrahyoid) [6]. Since Vaiman’s study, electroglottographic evaluation of the swallow did not provide normative data; the existing ones were based on videofluoroscopic studies [3].

The results of our study showed correlation of the surveys concerning swallowing dysfunction complaints with muscle dysfunction assessed by the speech therapist and SEMG. Giving the patient too many questionnaires is unnecessary. In our opinion, only DHI and SDS surveys should be used. According to its characteristics, DHI includes interalia emotional assessment, which gives a view on the patient’s approach and is helpful in monitoring the therapy. In this study, MTDg patients’ DHI results were significantly higher. The questionnaire containing questions about the quality of life indicated more severe symptoms in patients with MTDg than patients with atypical swallowing. This observation is consistent with the concept of both dysfunctions and the belonging of MTDg to dysphagia entity. Previous work by Krasnodębska pointed out that the SDS questionnaire characterised the severity of dysphagia objective symptoms and located the probable place of dysfunction. According to the literature, Swallowing Disorder Scale is more effective at detecting people with MTDg, compared to people with other causes of dysphagia, such as laryngeal paralysis or neurological diseases [19]. The material of this work included patients with the dysfunction of the initial swallowing stages (oropharyngeal phase); we think that is why SDS results did not correlate with patients’ subgroups. Among the questionnaires, SDS had the highest correlation ratio with SEMG results, especially questions 4–6, indicating the oropharyngeal cause of dysphagia [19]. Moreover, an interesting result of the analysis was the correlation of SDS questions 4–6 score with SEMG results of the submental muscles. The observation is similar to the results of Vaiman, who found smaller than normal amplitude and abnormal EMG signal shape of the submental muscles in patients with otolaryngological dysfunctions causing dysphagia [7]. Our research showed differences between the two examined groups of patients regarding SEMG results, firstly of submental and infrahyoid muscles during liquid and mash swallowing, and secondly, trapezius muscles during solid food swallowing. Observed differences may be more intense, due to different duration of the muscle activity during the swallow. Vaiman’s measurements during swallowing showed the longest activity of submental muscles, then infrahyoid muscles and the shortest of masseters. Dysfunctions observed by the speech therapist in persons with non-normative swallowing patterns were limited to the oral cavity. The speech therapist observed elongated processing of food and penetration from the oral cavity to the pharynx, while the time of the swallow was within the norm. SEMG observation revealed greater asymmetry of masseters during food formation in patients with atypical swallowing. Average muscle amplitude was comparable; we also did not find significant differences regarding maximum amplitude. As shown in the results, we found significant relation of logopaedic evaluation and amplitude values (average as well as maximum) of the infrahyoid muscles measured during swallowing of all kinds of food. Patients with more abnormalities in logopaedic examination had significantly higher infrahyoid muscle activity during swallowing. Observations are similar to those of Vaiman, who recorded increased signal value of infrahyoid muscles in patients with ENT diseases, compared with a normal population. In the ENT group, he also observed lower results than normal of submental muscles and normal results of masseters. In our opinion, the increased tension and functional asymmetry of infrahyoid muscles may influence the work of the larynx and, thus, disturb phonation. Moreover, the asymmetry in trapezius activity is connected with the patients’ discomfort. This could be distinctly different from the objective measurement of dysphagia. All patients in the study group reported swallowing difficulties and assessed them through questionnaires. The results of similar discomfort but of different strength in the two subgroups may be explained by various mechanisms of esophageal sensory function, which may account for the sensation of dysphagia without apparent delay in bolus transit [4].

Literature data point out laryngopharyngeal reflux to be the most common dysfunction coexisting with dysphagia [20,21]. Research of Kaufman shows the appearance of LPR in nearly 80% of patients with muscle tension dysphonia [22]. In our study we observed the dysfunction most commonly in the group of patients with MTDg (in 14 out of 32 people). We have also observed higher RSI values in patients with MTDg. In our opinion, this might be a result of a higher percent of patients with reflux diagnosis in the group and, hence, greater awareness of the symptoms. Comparable results of RFS in both groups of patients were puzzling and may indicate an undiagnosed and underestimated problem. The results of this work showed a correlation of infrahyoid muscle asymmetry with the RFS score. This observation may indicate a connection between hyoid bone position and reflux symptoms. Similar results were published by Angsuwarangsee [23]. The author found a significant relation between the thyrohyoid muscle tension (assessed by palpation) and the occurrence of reflux. This phenomenon is also observed in professional singers. As stated by Farneti, in singing, the physiological adjustments required to produce a more resonant voice alter, over time, the physiology of structures involved in swallowing by lowering the laryngotracheal axis and, thus, affecting the timing of swallowing [24]. In our observations, we found a significant correlation with infrahyoid muscle amplitude asymmetry and VHI results. Observations confirmed the effect of correct hyoid bone position on voice and swallowing disorders. In the context of co-occurrence of voice and swallowing dysfunction caused by abnormal muscle tension, it is reasonable to apply similar methods of rehabilitation. Functional voice therapy used successfully in dysphonias to restore muscle functionality may by prescribed for patients with MTDg [1,12,25,26].

## 5. Conclusions

Surface electromyography objectifies logopaedic examination of patients with swallowing difficulties. The results of this work showed that, apart from longer swallow, patients with MTDg differ from patients with non-normative swallowing pattern in the muscle activity measured by SEMG, abnormalities in logopaedic evaluation and the severity of complaints reported by patients (assessed in questionnaires). Evaluation of muscle amplitude symmetry should be included in the diagnostic protocol of SEMG.

## Figures and Tables

**Table 1:** Mean values of scores obtained from the questionnaires in subsequent patient groups. Pearson correlation of the questionnaires with the group type is shown.

	VHI	EAT-10	MST	DHI	SDS	RSI	RFS
Patients with non-normative swallowing pattern N = 26	48.9 (SD 30)	6.5 (SD 8)	0.2 (SD 0.6)	19 (SD 19)	13 (SD 8.5)	19 (SD 9)	4.2 (SD 2.7)
Patients with muscle tension dysphagia N = 32	35.8 (SD 34)	15 (SD 7.5)	1 (SD 1.3)	36 (SD 24)	14 (SD 9.6)	27 (SD 10)	4.6 (SD 4.1)
Correlation	No correltion	Average positive correlation	No correlation	Average positive correlation	No correlation	Weak positive correlation	No correlation

**Table 2:** Percentage of patients with abnormalities in logopaedic evaluation.

	Anatomic Evaluation							Functional Evaluation						
	Lips	Cheeks	Tongue	Occlusion	Dentition	Soft Palate	Hard Palate	Lips	Cheeks	Soft Palate	Tongue	Throat Posteriori Wall	Masseters	Temporo–Mandibular Joint
Patients with MTDg	13%	6.3%	81%	75%	94%	76%	0%	100%	38%	38%	94%	0%	50%	31%
Patients with atypical swallowing	69%	0%	100%	54%	100%	31%	0%	100%	31%	69%	100%	0%	69%	69%

**Table 3:** Mean values of the average and maximum amplitude of the examined muscles with the percent of asymmetry between muscle pairs (right and left muscle). * refers to the percent of patients in the subgroup who exceeded the value of 20% of muscle amplitude asymmetry.

			Liquid				Mash				Solid Food							
											Formation				Swallowing			
			Submental	Infrahyoid	Trapezius	Masseter	Submental	Infrahyoid	Trapezius	Masseter	Submental	Infrahyoid	Trapezius	Masseter	Submental	Infrahyoid	Trapezius	Masseter
Patients with MTDg	av ampl (uV)	av SD	185 33	156 24	134 10	145 23	185 34	150 17	132 10	153 34	191 23	161 18	141 15	215 36	207 34	163 30	139 11	191 40
	% asymm		3% * in 0%	2% * in 0%	2% * in 0%	10% * in 12%	4% * in 0%	3% * in 0%	2% * in 0%	14% * in 25%	6% * in 0%	5% * in 6%	1% * in 0%	14% * in 25%	8% * in 6%	5% * in 6%	1% * in 0%	12% * in 12%
	max ampl (uV)	av SD	640 201	416 140	293 63	373 167	665 291	365 97	281 79	400 199	713 281	532 166	368 115	976 368	869 253	496 149	336 94	802 357
	% asymm		11% * in 12%	11% * in 12%	13% * in 25%	36% * in 50%	14% * in 38%	10% * in 12%	7% * in 0%	27% * in 63%	27% * in 50%	23% * in 38%	4% * in 6%	28% * in 50%	22% * in 63%	15% * in 12%	6% * in 0%	42% * in 69%
Patients with non-normative swallowing pattern	av ampl (uV)	av SD	182 52	153 31	130 10	147 36	176 26	150 23	129 6	163 38	191 19	167 15	140 5	214 51	216 34	169 22	135 9	177 30
	% asymm		8% * in 0%	4% * in 0%	2% * in 0%	7% * in 8%	5% * in 0%	3% * in 0%	1% * in 0%	8% * in 8%	5% * in 0%	3% * in 0%	2% * in 0%	24% * in 23%	4% * in 0%	2% * in 0%	1% * in 0%	8% * in 0%
	max ampl (uV)	av SD	588 380	380 190	253 36	343 232	579 196	385 135	263 32	516 298	669 145	497 66	350 19	912 344	961 413	496 157	312 78	571 217
	% asymm		20% * in 31%	8% * in 0%	8% * in 8%	26% * in 23%	15% * in 8%	21% * in 15%	6% * in 0%	50% * in 23%	14% * in 8%	10% * in 8%	5% * in 0%	52% * in 23%	15% * in 15%	8% * in 0%	9% * in 0%	22% * in 15%

**Table 4:** The following table presents information in which situations increased asymmetry of muscles are observed for specific subpopulations. Muscle amplitude asymmetry values (average: av and maximum: max) during liquid (liq), mash and solid food swallowing and formation (solid s, solid f) are presented, depending on subgroup (patients with Atypical Swallowing (AtS), compared to patients with Muscle Tension Dysphagia (MTDg)), quantity of abnormalities in logopaedic examination, duration of swallowing and higher survey scores.

	Submental Asymmetry		Infrahyoid Asymmetry		Masseter Asymmetry		Trapezius Asymmetry	
	av	max	av	max	av	max	av	max
AtS, compared to MTDg	↑liq	↑liq			↑mash ↑solid s, f	↑mash ↑solid s, f		
More abn. in logop. exam.			↑liq ↑mash	↑liq ↑mash			↑liq ↑solid f	
Longer duration of swallow	↑mash ↑solid s	↑mash ↑solid s	↑mash ↑solid s	↑mash ↑solid s			↑mash ↑solid s	↑mash ↑solid s
Higher survey score	↑mash SDS, DHI, VHI	↑mash SDS, DHI, VHI	↑liq EAT10, VHI	↑liq EAT10, VHI			↑solid s SDS, DHI, EAT10, VHI	↑solid s SDS, DHI, EAT10, VHI
Higher RFS score			↑liq ↑mash	↑liq ↑mash				

## Data Availability

The datasets generated for this study are available on request to the corresponding author.
